# Integrated Analysis of Histophysiological Responses and Transcriptome–Metabolome Mechanisms in *Coelomactra antiquata* Under Ammonia Nitrogen Stress

**DOI:** 10.3390/ani16020192

**Published:** 2026-01-08

**Authors:** Dongming Huang, Sican Cai, Yongkang Hou, Hongli Qin, Yinyin Deng, Zhimin Li

**Affiliations:** 1 College of Fishery, Guangdong Ocean University, Zhanjiang 524088, China; huangdongming@stu.gdou.edu.cn (D.H.); 11111201ss@stu.gdou.edu.cn (S.C.); 2112101046@stu.gdou.edu.cn (Y.H.); qinhl@stu.gdou.edu.cn (H.Q.); dengyinyin@stu.gdou.edu.cn (Y.D.); 2 Guangdong Provincial Key Laboratory of Aquatic Animal Disease Control and Healthy Culture, Zhanjiang 524088, China

**Keywords:** *Coelomactra antiquata*, ammonia nitrogen stress, histophysiology, transcriptomics, metabolomics

## Abstract

*Coelomactra antiquata*, a marine bivalve of high nutritional and economic value, lacks comprehensive data on its toxic responses and adaptive mechanisms to ammonia nitrogen. This study integrated histophysiology, transcriptomics, and metabolomics to investigate its ammonia tolerance and molecular mechanisms, determining a 48 h LC_50_ of 99.06 mg/L and a sublethal concentration of 9.91 mg/L. After 48 h of sublethal ammonia stress, SOD, CAT, GLDH, and GS activities in gill and hepatopancreas significantly increased, with notable changes in MDA, Gln, and urea contents, confirming disruption of antioxidant defense and nitrogen metabolism homeostasis. Tissue sections revealed irreversible histopathological damage to key tissues. Omics analyses identified 7823 differentially expressed genes (DEGs) and 737 differentially expressed metabolites (DEMs) in hepatopancreas. DEGs were enriched in metabolic pathways and multiple immune-related signaling pathways (e.g., NF-kappa B, RIG-I-like receptor), while DEMs were primarily involved in processes such as protein digestion/absorption, aminoacyl-tRNA biosynthesis, and amino acid metabolism. Research data indicate that ammonia nitrogen stress primarily regulates the antioxidant function and nitrogen metabolism homeostasis of *C. antiquata* by activating multiple immune- and metabolism-related pathways. This first systematic multi-omics study elucidates *C. antiquata*’s tolerance to ammonia nitrogen and its molecular responses, filling a gap in environmental toxicology research for sustainable aquaculture and genomic studies.

## Simple Summary

*Coelomactra antiquata* is a type of marine shellfish that is nutritious and economically valuable. However, little is known about how it responds to ammonia nitrogen, a common pollutant in aquatic environments, and how it adapts to this stress. This study aimed to determine *C. antiquata*’s ability to tolerate ammonia nitrogen and the internal processes that help it cope. We first determined *C. antiquata*’s ammonia tolerance, finding LC_50_-48 h (99.06 mg/L) and SC (9.91 mg/L). When exposed to a 99.06 mg/L concentration for 48 h, *C. antiquata*’s gill and hepatopancreas (a key metabolic organ) showed changes in important enzymes and substances, which disrupted its ability to fight cell damage and balance nitrogen in the body. The tissues also suffered permanent damage, and further analysis revealed changes in thousands of genes and hundreds of small molecules related to metabolism, immunity, and detoxification. These findings explain how *C. antiquata* deals with ammonia nitrogen stress and provide useful information for protecting its survival in aquaculture, supporting the sustainable development of the industry.

## 1. Introduction

*Coelomactra antiquata*, belonging to the phylum Mollusca, class Lamellibranchia, order Veneroida, family Mactridae, and genus *Coelomactra*, is a large benthic clam [1]. It can reach a shell length of up to 15 cm, inhabits sandy substrates from the lower intertidal zone to 20 m depth, and is widely distributed along China’s coasts, with the highest population density in Fujian Province [2]. *C. antiquata* has high market value. Besides being a long-renowned precious delicacy, it also possesses significant medical and health-care value, making it a rare shellfish with both edible and medicinal properties [3]. However, due to overfishing and habitat destruction, the population of this species declined sharply from the late 1980s to the 1990s [4]. Habitat degradation caused by various adverse environmental conditions directly affects the survival, growth, and reproduction of *C. antiquata*, leading to a simultaneous reduction in the size of its natural populations and the yield of artificial cultivation [1,5,6]. Among these key environmental factors, ammonia nitrogen pollution is one of the important causes of these problems, as it exhibits potential toxicity to benthic mollusks [7]. Therefore, studying the stress response of *C. antiquata* to ammonia nitrogen and its regulatory mechanisms can provide crucial theoretical support for the sustainable development of a healthy aquaculture industry for *C. antiquata* and holds significant practical value.

Ammonia, a major toxicant in aquaculture water, has become a key bottleneck for aquaculture development [8]. It primarily originates from animal excreta, industrial/municipal wastewater, and the decomposition of organic matter, like uneaten feed [9,10]. Therefore, in intensive aquaculture systems, ammonia tends to accumulate, which may cause acute or chronic poisoning in aquatic animals during the cultivation process [11]. Total ammonia nitrogen (TAN) exists in ionic (NH_4_^+^) and unionized (NH_3_) forms [12]. Because unionized ammonia can penetrate cell membranes and diffuse, high concentrations of environmental ammonia can trigger a series of adverse reactions in fish, shrimp, and mollusks during aquaculture [13,14], such as respiratory dysfunction, metabolic disorders, immunosuppression, oxidative stress, and tissue damage [15,16,17,18]. For instance, sublethal ammonia concentrations reduce *Haliotis midae* survival and growth [19] while disrupting lysosomal integrity and triggering gill cell apoptosis in *Ruditapes philippinarum* [20]. Ammonia nitrogen stress also induces excessive reactive oxygen species (ROS) production, causing tissue, protein, and DNA damage [21,22,23]. In *Solenaia oleivora*, initial ammonia exposure activates antioxidant defenses, but prolonged exposure overwhelms these mechanisms, leading to ROS accumulation, malondialdehyde (MDA) buildup, and lipid peroxidation [24]. Antioxidant enzymes alleviate oxidative stress by neutralizing reactive oxygen species such as hydrogen peroxide while maintaining cellular homeostasis [25,26,27]. Elevated ammonia concentrations also inhibit ammonia excretion in aquatic animals, resulting in the accumulation of endogenous and exogenous ammonia [28]. Specific metabolic enzymes, however, detoxify ammonia by converting it into non-toxic or low-toxicity substances for excretion [29,30]. The widespread use of high-density intensive aquaculture systems has resulted in excessive ammonia nitrogen in aquaculture water, which has become a prominent environmental problem [31]. Although the toxic effects of ammonia have been extensively studied in mollusks, data on the mechanisms of ammonia toxicity in *C. antiquata* remain relatively limited. Furthermore, *C. antiquata* differs significantly from other bivalves in ecological habits, aquaculture models, and other aspects [2,4,7]. Existing research findings cannot be directly applied, which results in the uniqueness of ammonia nitrogen stress challenges faced by *C. antiquata*. 

With the rapid development of high-throughput sequencing technologies, omics techniques such as transcriptomics and metabolomics have been widely applied to explore the intrinsic mechanisms of organisms in responding to environmental changes or pollution [32]. High-sensitivity transcriptomics is used to reveal changes at the upstream RNA level, thereby investigating differentially expressed genes and their related functions, while metabolomics is employed to uncover alterations in downstream differentially expressed metabolites in organisms under external stress [33,34]. Currently, transcriptomic and metabolomic technologies have been applied in studies on bivalves such as *Corbicula fluminea* [35], *Mizuhopecten yessoensis* [36], *Meretrix petechialis* [37], and *Ruditapes philippinarum* [20]. Single-omics technologies have certain limitations in research, while multi-omics analysis methods combine data from two or more omics studies. Compared with single-omics approaches, the integrated analysis of transcriptomics and metabolomics is better suited for comprehensive evaluation and verification of environmental factors. Relevant studies have been applied to ammonia nitrogen stress in *Solenaia oleivora* [24], low-temperature stress in *Acanthopagrus schlegelii* [38], hypoxia stress in *Pelteobagrus fulvidraco* [39], and metal stress in *Pirata subpiraticus* [40]. To explore the molecular response mechanisms of *C. antiquata* under ammonia nitrogen stress, this study adopted an integrated analysis of transcriptomics and metabolomics. Transcriptomics was used to screen globally differentially expressed genes, and metabolomics was combined to systematically reveal differential metabolic pathways, fully leveraging the high-throughput and high-sensitivity advantages of both techniques [33,34].

Therefore, this study aimed to investigate the acute toxic effects of ammonia nitrogen stress on *C. antiquata*, analyze structural changes in its gill and hepatopancreas tissues under ammonia nitrogen stress through histological observations, clarify the regulatory patterns of antioxidant metabolism and detoxification mechanisms in this species, and deeply explore its molecular mechanisms in response to ammonia nitrogen stress via integrated transcriptomic and metabolomic analyses. The experimental results not only revealed the immune regulatory mechanisms of *C. antiquata* under ammonia nitrogen stress but also laid an important foundation for subsequent studies on molecular mechanisms.

## 2. Materials and Methods

### 2.1. Experimental Animals

Healthy *C. antiquata* (shell length: 7.70 ± 0.2 cm, shell width: 4.08 ± 0.1 cm, shell height: 6.32 ± 0.2 cm, weight: 86.35 ± 10.5 g) used in this study were purchased from the seafood market in Zhanjiang City, Guangdong Province. Prior to the experiment, all clams were acclimated in water tanks for 1 week under continuous aeration. The experimental *C. antiquata* were fed with *Chlorella* sp. twice a day, and 1/2 of the tank water was changed daily. The acclimation conditions were maintained at a temperature of (25 ± 1) °C, salinity of 27 ± 1‰, and pH 8.0 ± 0.1.

### 2.2. Acute Toxicity Tests

Based on the results of preliminary experiments, we set six treatment groups with ammonia nitrogen concentrations of 0, 20, 40, 80, 120, and 160 mg/L. Each concentration group had three replicate groups, and 15 *C. antiquata* were placed in each replicate group. Analytical-grade ammonium chloride was used to prepare the ammonia nitrogen solution. During the toxicity test, the ammonia nitrogen concentration was measured every 6 h at a wavelength of 420 nm using Nessler’s reagent spectrophotometry [41]. If significant fluctuations in ammonia nitrogen concentration were observed, the target concentration was maintained by supplementing the test solution. Additionally, water was changed every 12 h for further adjustment to ensure the concentration remained stable. The experiment was conducted in 120 L water tanks under the following conditions: temperature (25 ± 1) °C, salinity 27 ± 1‰, and pH 8.0 ± 0.1. No feed was provided during the experiment, and micro-aeration was maintained. Mortality was observed regularly, and dead individuals were removed promptly. The number of dead individuals was recorded at 6, 12, 24, and 48 h. The death of *C. antiquata* was determined by relaxation of the adductor muscle (inability to close the shell), complete extension and relaxation of the pedal foot, and failure to retract spontaneously.

The mass of ammonium chloride required was calculated based on the conversion relationship between ammonia nitrogen (N) and ammonium chloride. The relative atomic mass of ammonia nitrogen (N) is 14, and the relative molecular mass of ammonium chloride (NH_4_Cl) is 53.5. Assuming the preparation of an ammonia nitrogen solution with a volume of V (L) and a concentration of C (mg/L), the formula for calculating the required mass of ammonium chloride m (mg) is

$$m=\frac{C\times V\times 53.5}{14}$$

Data were processed using SPSS 23.0 software to establish a regression equation. The 48 h median lethal concentration (48 h LC_50_) was calculated using the linear regression method, and the safe concentration (SC) was derived using the following formula [42]: SC = 0.1 × 48 h LC_50_.

### 2.3. Ammonia Stress and Sample Collection

The stress experiment was divided into two groups: control group (C) and treatment group (T), with three parallel groups per group and 15 *C. antiquata* per parallel group. The control group was not exposed to ammonia nitrogen, while the treatment group was subjected to the 48 h LC_50_ concentration of ammonia nitrogen (99.06 mg/L). Gill and hepatopancreas tissues were collected at 0, 6, 12, 24, and 48 h of ammonia nitrogen stress; stored at −80 °C; and used for subsequent analyses of enzyme activities, transcriptomics, and metabolomics. To avoid sample contamination and RNA degradation, both sampling tools and the sampling environment were treated with 75% alcohol and exogenous RNase eliminator prior to sampling.

### 2.4. Measurements of Physiological Parameters

In this study, the double-antibody one-step sandwich enzyme-linked immunosorbent assay (ELISA) was employed to detect relevant physiological indicators in the gills and hepatopancreas of *C. antiquata*. The gill and hepatopancreatic tissues of *C. antiquata* were thawed on ice, weighed, placed into a homogenizer, and mixed with PBS buffer (pH 7.4). All operations were performed on ice. After the samples were fully homogenized using a homogenizer, they were centrifuged at 2500 r/min at 4 °C for 20 min. The supernatant was collected for subsequent detection. All experimental steps were strictly conducted in accordance with the instructions in the ELISA kit provided by Shanghai Enzyme-Linked Biotechnology Co., Ltd (Shanghai, China). The microplate reader was set to a wavelength of 450 nm for absorbance measurement, and the correlation coefficient (R value) between the linear regression of the standard and the expected concentration was ≥0.99. Immune function-related indicators included superoxide dismutase (SOD) activity, catalase (CAT) activity, and malondialdehyde (MDA) content; ammonia metabolism-related indicators included glutamate dehydrogenase (GLDH) activity, glutamine synthetase (GS) activity, glutamine (Gln) content, and urea content.

### 2.5. Histological Observation

*C. antiquata* were exposed to the 48 h LC_50_ concentration of ammonia nitrogen. Gill and hepatopancreas tissues were collected at 0, 12, 24, and 48 h post-exposure, fixed in paraformaldehyde solution for 24 h, washed repeatedly with 70% ethanol, dehydrated through a graded ethanol series, and cleared with xylene to prepare for paraffin infiltration. The tissues were then embedded in molten paraffin to form uniform paraffin blocks. After solidification, the blocks were sectioned into 5–10 μm slices using a microtome, mounted on glass slides, deparaffinized, stained with hematoxylin and eosin, dehydrated again, cleared, and coverslipped. Histopathological changes were observed under an optical microscope and photographed for documentation.

### 2.6. Transcriptomic Analysis

Three hepatopancreas tissue samples were randomly selected from each time point in both the control and treatment groups. Total RNA was extracted using Trizol reagent. The concentration and purity of RNA were assessed using a NanoDrop 2000 spectrophotometer (Thermo Fisher Scientific, Waltham, MA, USA), and RNA integrity was determined via 1% agarose gel electrophoresis. A total of 24 sequencing libraries were constructed following Illumina’s standard RNA-seq library preparation protocol [43]. After quality assessment, the libraries were sequenced on an Illumina NovaSeq6000 system by Gene Denovo Biotechnology Co (Guangzhou, China). The raw sequencing reads were submitted to the Sequence Read Archive (SRA) at NCBI (GenBank project accession PRJNA1303244) using FileZilla Client. To ensure the quality of sequencing data, the raw data from the above 24 transcriptome libraries needed to be filtered to reduce the impact of invalid data on the analysis. Fastp (v0.18.0) [44] was used for quality control of the raw reads generated by the sequencer, aiming to filter out low-quality data and obtain high-quality reads. The methods for filtering raw reads included removing reads containing adapters, reads with an N (undetermined nucleotide) content exceeding 10%, reads consisting entirely of adenine (A) bases, and low-quality reads (where bases with a quality value Q ≤ 20 account for more than 50% of the entire read). The filtered reads are referred to as high-quality reads (HQ reads).

Differential gene expression analysis between the control and treatment groups was performed using DESeq2 software [45]. This included gene normalization, calculation of *p*-values, and multiple hypothesis testing correction to obtain false discovery rate (FDR) values. Genes with a fold change (FC) > 2, *p* ≤ 0.05, and FDR < 0.05 were considered differentially expressed genes (DEGs). Subsequently, Gene Ontology (GO) term and Kyoto Encyclopedia of Genes and Genomes (KEGG) pathway enrichment analyses were conducted to identify significantly enriched regulatory pathways. Then, Short Time-series Expression Miner (STEM) software was used to perform trend analysis of samples [46]. By analyzing the expression profiles of DEGs at 4 time points (different stages after ammonia nitrogen stress), the dynamic characteristics of gene abundance in *C. antiquata* during ammonia nitrogen stress were clarified. Next, weighted gene co-expression network analysis (WGCNA) was applied to cluster genes with similar expression patterns, construct co-expression modules, and analyze the associations between modules and specific traits or phenotypes. Statistical analysis of DEGs was performed using R language [47]. Cytoscape (v3.7.1) [48] was employed to construct gene co-expression networks, visualizing hub genes and their interaction relationships.

### 2.7. Metabolomic Analysis

Twenty-four hepatopancreas tissue samples were processed using a standard metabolomics protocol for untargeted liquid chromatography–mass spectrometry (LC-MS) analysis. Precisely 100 mg of each sample ground in liquid nitrogen was extracted with 1 mL of pre-chilled methanol–acetonitrile–water (2:2:1, *v*/*v*). After vortex mixing, the samples were ultrasonically treated twice at low temperature for 30 min each, then kept at −20 °C for 60 min to promote protein precipitation. The supernatant was collected via centrifugation (14,000× *g*, 4 °C, 20 min) and dried under vacuum. Before mass spectrometry analysis, the dried samples were reconstituted with 100 μL of acetonitrile–water (1:1, *v*/*v*), vortexed, centrifuged again (14,000× *g*, 4 °C, 15 min), and the supernatant was injected for analysis. To monitor experimental quality, quality control (QC) samples were prepared by mixing equal volumes of all test samples, used to assess instrument stability and system reliability throughout the analysis.

Raw metabolomic data were processed using ProteoWizard v3.0 software for filtering, peak identification, extraction, and missing-value imputation. Metabolite annotation was performed using Gene Ontology (GO) and Kyoto Encyclopedia of Genes and Genomes (KEGG) databases. Multivariate analyses, including principal component analysis (PCA), orthogonal partial least squares discriminant analysis (OPLS-DA), and loading plots, were used to exclude outliers and screen for potential biomarkers. Differentially expressed metabolites (DEMs) were identified using variable importance in projection (VIP) ≥ 1 and *p* < 0.05. KEGG pathway enrichment analysis was conducted via Fisher’s exact test, with results visualized to highlight significantly enriched metabolic pathways.

### 2.8. Integrated Transcriptomic and Metabolomic Analysis

Differentially expressed genes from transcriptomics and differentially expressed metabolites from metabolomics were mapped to Kyoto Encyclopedia of Genes and Genomes (KEGG) pathways. DEGs and DEMs in common pathways were screened and analyzed. Based on KEGG enrichment results, heat maps were used to visualize the potential correlations between DEG and DEM expressions, revealing gene-metabolite changes induced by ammonia nitrogen stress.

### 2.9. Quantitative Real-Time PCR (qRT-PCR) Validation of DEGs

To validate the Illumina sequencing data, 15 key immune-related genes were selected for qRT-PCRto verify their expression levels. Gene-specific primers were designed using Primer Premier v5.0, with β-actin serving as the reference gene; detailed primer information is provided in Table 1. RT-qPCR was performed on a Light Cycler 96 system in a 20.0 μL reaction mixture containing 10 μL of 2×PerfectStar^®^ Green PCR Super Mix, 6.4 μL of nuclease-free water, and each primer (concentration adjusted to protocol). The PCR program was as follows: preincubation at 94 °C for 30 s; 40 cycles of three-step amplification (94 °C for 5 s, 60 °C for 15 s, 72 °C for 10 s); melting curve analysis (95 °C for 60 s, 65 °C for 30 s, 95 °C for 1 s); cooling at 40 °C for 60 s. Each sample was analyzed in triplicate on Light Cycler 96, and relative gene expression was calculated using the 2^−ΔΔCt^ method, with each reaction repeated three times.

### 2.10. Statistical Analysis

In this study, one-way analysis of variance (ANOVA) was used for statistical analysis of different enzyme activity parameters in *C. antiquata* under ammonia nitrogen stress. All data and graphs were analyzed and generated using SPSS 23.0 and Excel software, with data presented as mean ± standard deviation. Significance levels are denoted as * *p* < 0.05 (significant), ** *p* < 0.01 (highly significant), *** *p* < 0.001 (extremely significant).

## 3. Results

### 3.1. Lethal Effects of Ammonia Nitrogen Stress on C. antiquata

**Figure 1:**
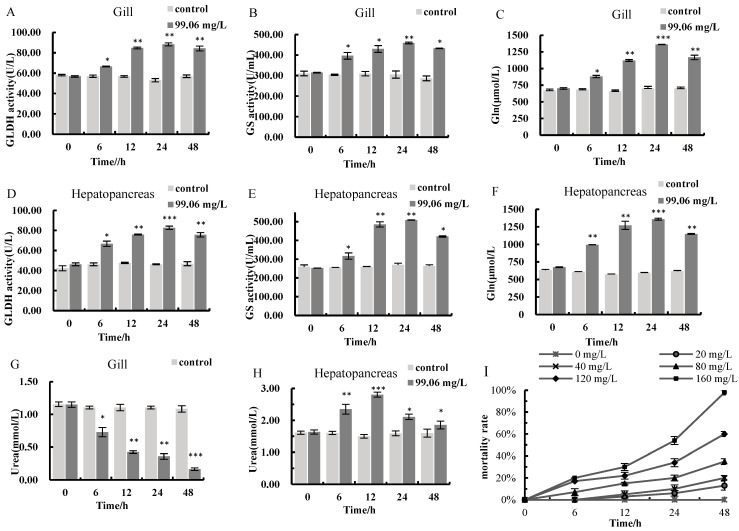
(**A**–**H**): The changes in antioxidant indexes in gills and hepatopancreas. (**I**): The mortality rate of *C. antiquata* under differing ammonia nitrogen stress. Asterisks denote statistical differences between control and treatment groups at the same time point: * *p* < 0.05 (significant), ** *p* < 0.01 (highly significant), *** *p* < 0.001 (extremely significant).

As depicted in Figure 1I, no mortalities were recorded in the control group (0 mg/L) within 48 h. In experimental groups, mortality increased monotonically with the ammonia nitrogen concentration. Time-dependent mortality escalation was also observed: at 120 mg/L, mortality rose from 7% at 6 h to 60% at 48 h, with a steep post-6 h increase (Figure 1I). SPSS 23.0 analysis yielded a 48 h LC_50_ of 99.06 mg/L and a safe concentration (SC) of 9.91 mg/L for *C. antiquata*. Corresponding non-ionic ammonia concentrations were 8.915 mg/L (LC_50_) and 0.892 mg/L (SC), with a 95% confidence interval for total ammonia nitrogen (TAN) of 82.38–119.87 mg/L.

### 3.2. Physiological Responses of C. antiquata to Ammonia Nitrogen Stress

During 0–48 h of ammonia nitrogen stress, the activities of glutamate dehydrogenase (GLDH), glutamine synthetase (GS), and glutamine (Gln) content, which are indices related to nitrogen metabolism in *C. antiquata*, exhibited a trend of first increasing and then decreasing in both gills and hepatopancreas. All indices reached their maximum values at 24 h, decreased at 48 h, but remained significantly higher than those in the control group *(p* < 0.01, *p* < 0.05) (Figure 1A–F). Urea content showed tissue-specific responses: in the hepatopancreas, it peaked at 12 h (2.81 mmol/L) and decreased to 1.86 mmol/L at 48 h, still significantly higher than the control group (*p* < 0.05); in gill tissue, however, urea content decreased significantly (*p* < 0.05, *p* < 0.001) from 1.15 mmol/L to 0.17 mmol/L (Figure 1G,H). 

**Figure 2:**
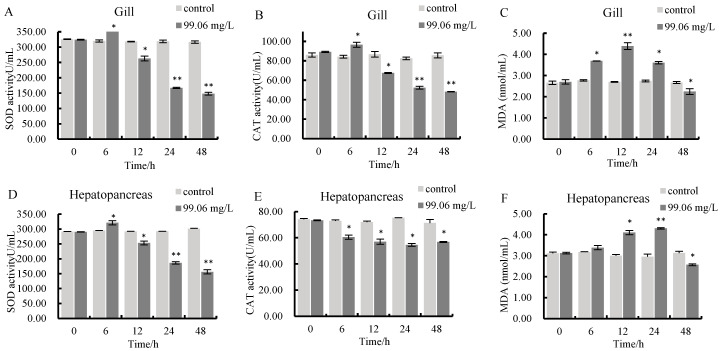
(**A**–**F**): Changes in antioxidant indexes in gills and hepatopancreas. Asterisks denote statistical differences between control and treatment groups at the same time point: * *p* < 0.05 (significant), ** *p* < 0.01 (highly significant).

During 0–48 h of ammonia nitrogen stress, the activities of superoxide dismutase (SOD) and catalase (CAT), as well as the content of malondialdehyde (MDA), which serve as indices related to antioxidant capacity in *C. antiquata*, showed a trend of first increasing and then decreasing in both gills and hepatopancreas. At 48 h, all indices were significantly lower than those in the control group (*p* < 0.01, *p* < 0.05), with higher antioxidant enzyme activities in the hepatopancreas than in the gills. Specifically, SOD activity peaked at 6 h in both tissues (significantly higher than the control group, *p* < 0.05) and was significantly lower than the control group at 48 h (*p* < 0.01) (Figure 2A,D). CAT activity exhibited tissue specificity: it peaked at 6 h in gills and was significantly lower than the control group at 48 h (*p* < 0.05), while in the hepatopancreas, it decreased significantly within 0–6 h (*p* < 0.05) (Figure 2B,E). MDA content first increased and then decreased in both tissues, peaking at 12 h in gills and 24 h in the hepatopancreas, with both being significantly lower than the control group at 48 h (*p* < 0.05) (Figure 2C,F).

### 3.3. Histological Observations

**Figure 3:**
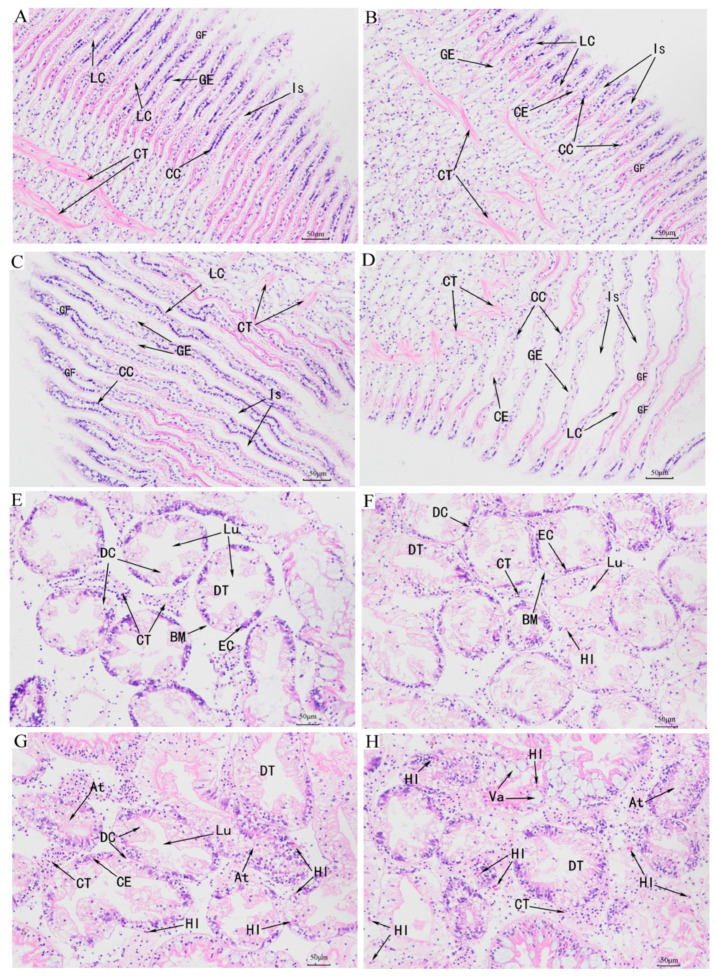
Time–course histopathological changes in gills and hepatopancreas of *C. antiquata* under ammonia stress. (**A**): Gill of the control group; (**B**–**D**): gill after 12, 24, and 48 h of ammonia nitrogen stress; (**E**): hepatopancreas of the control group; (**F**–**H**): hepatopancreas after 12, 24, and 48 h of ammonia nitrogen stress. LC: lateral cilia; GF: gill filament; GE: gill epithelium; CC: columnar cell; Is: inter—lamellar space; CT: connective tissue; CE: cilia exfoliation; DT: digestive tubule; DC: digestive cell; Lu: lumen; EC: epithelial cell; BM: basement membrane; HI: hemolytic infiltration; Va: vacuole; At: atrophy. Bar = 50 µm (×200).

Pathological sections of the gill and hepatopancreas tissues of *C. antiquata* under ammonia nitrogen stress are shown in Figure 3. In the healthy state, gill filaments (GF) were densely arranged in a comb-like architecture, covered by gill epithelium (GE) composed of a single layer of orderly columnar cells (CC), with frontal cilia, lateral cilia (LC), and posterior cilia distributed on the surface (Figure 3A). After 12 h of exposure to an ammonia nitrogen solution with molecular ammonia at 8.915 mg/L, partial cilia exfoliation (CE) occurred, while columnar cells remained structurally intact and tightly arranged (Figure 3B). At 24 h post-exposure, cilia exfoliation (CE) intensified, inter-lamellar spaces (Is) expanded, and connective tissue (CT) showed mild atrophy (Figure 3C). By 48 h, ciliary structures were almost completely lost, inter-lamellar spaces (Is) further widened, gill filaments became sparsely arranged, and connective tissue medial to columnar cells atrophied and disappeared (Figure 3D).

In the absence of ammonia nitrogen stress, digestive tubules (DTs) of the hepatopancreas exhibited clear lumens (Lu), with the outer layer of digestive tubules surrounded by a basal membrane (BM) and the inner layer lined with epithelial cells (ECs) (Figure 3E). After 12 h of exposure to 8.915 mg/L molecular ammonia, the tissue architecture showed no obvious alterations, except for minimal hemolymphocyte infiltration (HI) (Figure 3F). At 24 h, glandular tubules swelled with narrowed lumens, reduced inter-tubular spaces, partial exfoliation of epithelial cells, intensified hemolymphocyte infiltration, and atrophy of digestive cells (Figure 3G). By 48 h, extensive necrosis and exfoliation of epithelial cells occurred, digestive cells showed severe atrophy, digestive lumens nearly vanished, massive hemolymphocyte infiltration was observed, and vacuolization (Va) developed (Figure 3H).

### 3.4. DEG Identification After Ammonia Nitrogen Stress in C. antiquata

Twenty-four transcriptomic libraries were constructed from three biological replicates of control and treatment groups (99.06 mg/L) at 6 h, 12 h, 24 h, and 48 h, followed by Illumina NovaSeq6000 sequencing. A total of 982,706,716 raw reads were generated, of which 99.87% were filtered into high-quality (HQ) reads. After rRNA depletion, 956,928,440 valid reads were obtained, with Q20 > 98.04%, Q30 > 96.01%, and GC content ranging from 38.38% to 39.99% (Table 2). Valid reads from each library were aligned to the genome of *C. antiquata*. The alignment rates of the 24 transcriptome libraries were remarkably high, ranging from 76.29% to 85.14% (Table 3). Mapping of valid reads to the *C. antiquata* genome showed high mapping rates (76.29–85.14%) across all 24 libraries. This sequencing identified 23,652 genes, including 21,437 known genes (detection rate: 87.17%) and 2215 novel genes (accounting for 8.26% of the reference genome). The complete clean reads were uploaded to the NCBI database with accession number PRJNA1303244.

**Figure 4:**
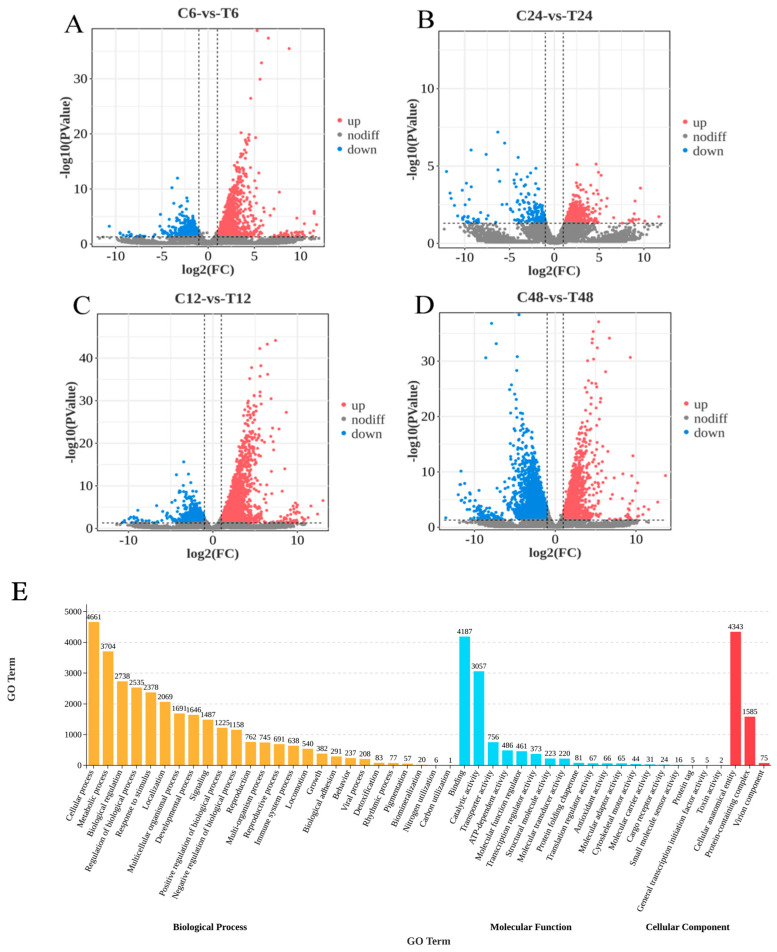
(**A**–**D**): volcanic map of DEGs. Note: red and blue dots represent upregulated and downregulated DEGs, while gray dots represent non-DEGs. (**E**): GO classification secondary bar chart. Note: the vertical coordinate of the graph is the GO term, and the horizontal coordinate is the number of target genes enriched in that GO term. The longer the bar, the higher the number of target genes enriched.

Comparisons between control and treatment groups at each time point (C6-vs-T6, C12-vs-T12, C24-vs-T24, C48-vs-T48) identified 2103 (1579 upregulated, 524 downregulated) (Figure 4A), 4030 (3213 upregulated, 817 downregulated) (Figure 4B), 843 (644 upregulated, 199 downregulated) (Figure 4C), and 5020 (2051 upregulated, 2969 downregulated) (Figure 4D) differentially expressed genes, respectively. In total, 7823 DEGs (33.07% of all detected genes) were identified under ammonia nitrogen stress. Among these, 66 DEGs showed consistent differential expression across all time points (Supplementary Table S1). KEGG enrichment analysis of the 66 DEGs revealed immune-related pathways (e.g., Toll-like receptor signaling pathway, NF-κB signaling pathway, Toll and Imd signaling pathway, NOD-like receptor signaling pathway, TNF signaling pathway), metabolism-related pathways (e.g., glycine, serine, and threonine metabolism; porphyrin metabolism; metabolic pathways), and signal transduction pathways (e.g., HIF-1 signaling pathway, PPAR signaling pathway, Rap1 signaling pathway), excluding human disease-related pathways.

### 3.5. Functional and Pathway Enrichment Analysis of DEGs Under Ammonia Nitrogen Stress

**Figure 5:**
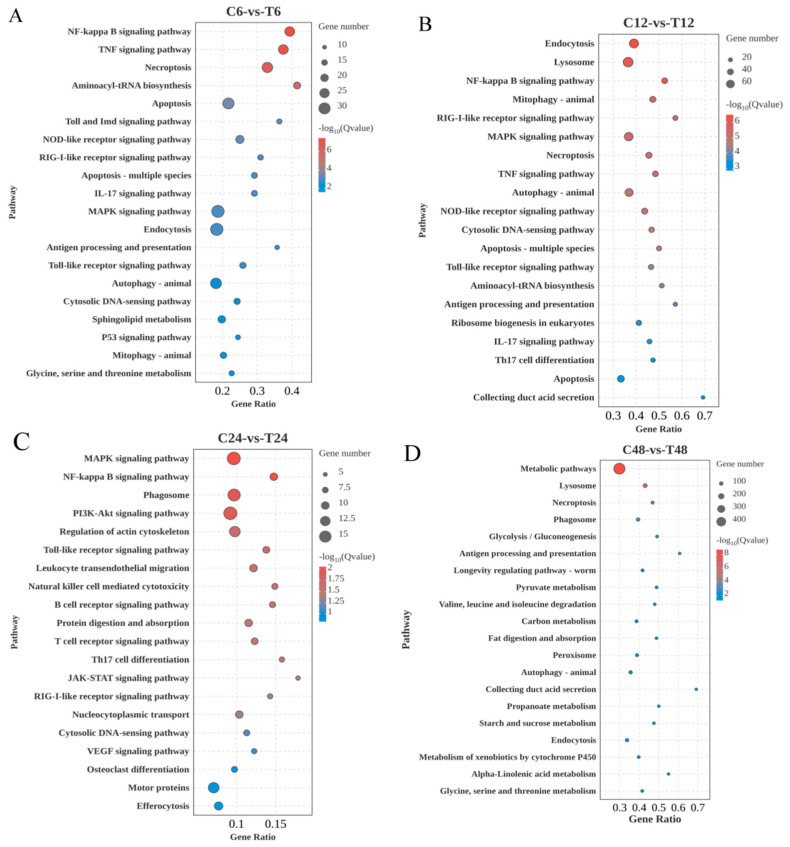
(**A**–**D**): Bubble diagram of significant enrichment of differential gene KEGG. Note: the horizontal coordinate is gene ratio, i.e., the number of target gene sets enriched in the current pathway/the number of species enriched in the current pathway; the vertical coordinate is pathway, where each bubble represents a pathway, the size of the bubble represents the number of genes contained in the pathway, and the color of the bubble represents the degree of significance of enrichment of the pathway.

GO enrichment analysis was performed on 7823 DEGs to explore their potential functions (Figure 4E). The analysis categorized DEGs into three main ontologies: biological process (BP), molecular function (MF), and cellular component (CC). In the BP category, genes were predominantly enriched in cellular process, metabolic process, biological regulation, and response to stimulus. The MF category was dominated by genes involved in binding, catalytic activity, and transporter activity. In the CC category, genes were mainly associated with cellular anatomical entities and protein-containing complexes. KEGG pathway enrichment analyses of DEGs from C6-vs-T6, C12-vs-T12, C24-vs-T24, and C48-vs-T48 identified 344, 358, 298, and 367 activated or inhibited pathways, respectively. Bubble plots were generated based on the top 20 enriched pathways in each group. At 6 h, significantly enriched immune-related pathways included the NF-κB signaling pathway, NOD-like receptor signaling pathway, and RIG-I-like receptor signaling pathway; apoptosis-related pathways included apoptosis and apoptosis—multiple species; and genetic information processing included aminoacyl-tRNA biosynthesis (Figure 5A). At 12 h, enriched immune pathways comprised the cytosolic DNA-sensing pathway, NOD-like receptor signaling pathway, and RIG-I-like receptor signaling pathway; cell process-related pathways included endocytosis, lysosome, and mitophagy—animal (Figure 5B). At 24 h, immune-related pathways, such as the NF-κB signaling pathway, Toll-like receptor signaling pathway, and natural killer cell-mediated cytotoxicity, were significantly enriched; cell process pathways included phagosome, regulation of actin cytoskeleton, and leukocyte transendothelial migration (Figure 5C). At 48 h, metabolism-related pathways dominated, including metabolic pathways, glycolysis/gluconeogenesis, pyruvate metabolism, and fat digestion and absorption; cell process pathways included lysosome and phagosome (Figure 5D).

### 3.6. Trend Analysis of DEGs

**Figure 6:**
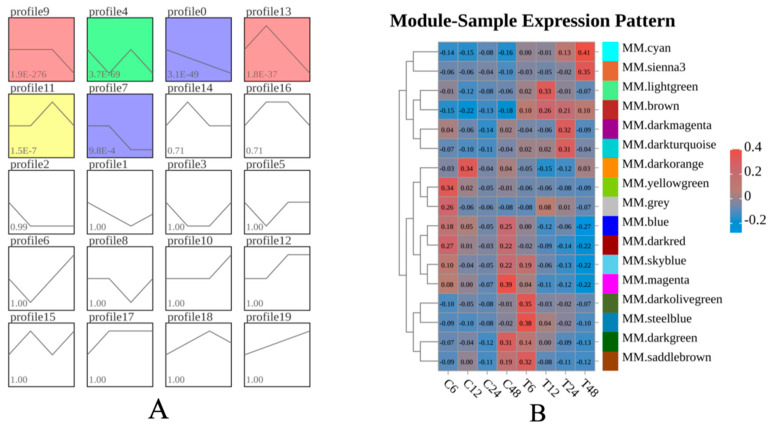
(**A**): Differential gene expression trend. The trend blocks with color indicate significantly enriched trends (*p* < 0.05), modules with similar trends have the same color, and each inflection point represents a group of sample data. (**B**): module-sample expression pattern. Positive and negative values represent the relative upregulation and downregulation of module expression levels; red represents high expression, and blue represents low expression.

To gain insight into the dynamic changes in DEGs across four time periods, DEGs with identical expression patterns were grouped into the same module using STEM, resulting in a total of 20 modules (Figure 6A). Among these, six modules (profile9, profile4, profile0, profile13, profile11, and profile7) showed significant enrichment. Profile 9, profile 0, and profile 7 exhibited a downward trend after ammonia nitrogen stress, while profile 13 and profile 11 displayed a trend of first increasing and then decreasing. DEGs in these modules were primarily expressed in substance metabolism-related pathways and immune/inflammatory signaling pathways, such as metabolic pathways, tryptophan metabolism, phenylalanine metabolism, galactose metabolism, NF-κB signaling pathway, RIG-I-like receptor signaling pathway, cytosolic DNA-sensing pathway, NOD-like receptor signaling pathway, and Toll and Imd signaling pathway.

### 3.7. WGCNA Analysis

**Figure 7:**
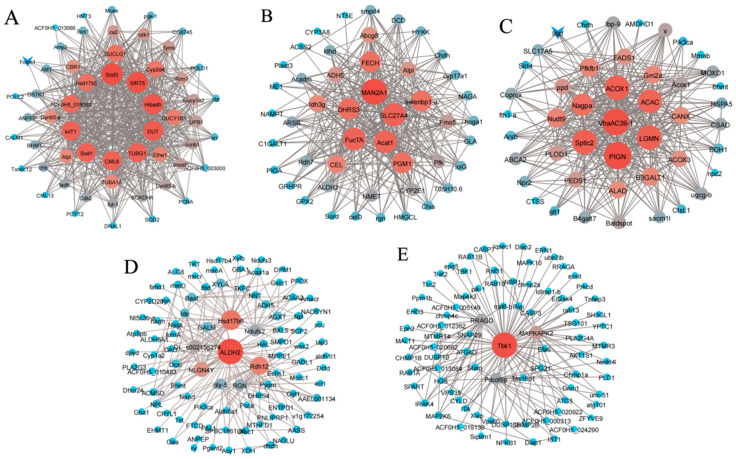
DEG co-expression network. (**A**): Darkred module. (**B**): Magenta module. (**C**): Cyan module. (**D**): Blue module. (**E**): Brown module. Each point in the graph represents a gene, and each line represents the existence of a regulatory relationship between the points. The node color and size can be artificially defined by gene abundance or connectivity; the darker and larger the node color, the higher the abundance and the stronger the connectivity.

WGCNA was performed on 7823 DEGs with a power value of 8, yielding 17 modules. Module eigengenes were used to represent the expression patterns of module genes across samples, and a heatmap of sample expression patterns was generated (Figure 6B). The cyan and sienna3 modules showed continuously upregulated expression levels, while the blue, darkred, skyblue, and magenta modules exhibited continuously downregulated expression. In contrast, the lightgreen, brown, darkmagenta, darkturquoise, darkolivegreen, steelblue, and saddlebrown modules displayed a trend of first upregulation followed by downregulation. DEGs in these modules were mainly enriched in substance metabolism-related pathways and immune/inflammatory signaling pathways, including metabolism of xenobiotics by cytochrome P450, MAPK signaling pathway, NF-κB signaling pathway, RIG-I-like receptor signaling pathway, D-Amino acid metabolism, and efferocytosis. Gene co-expression networks were constructed for the darkred, magenta, cyan, blue, and brown modules, as shown in Figure 7. A total of 54 hub genes were identified, including *Sptic2*, *FADS1*, *CSAD*, *Sod3*, *Hibadh*, *GUCY1B1*, *Tbk1*, *RPAGD*, *ALDH2*, *Rdh12*, *Hsd17b6*, *MAN2A1*, *FECH*, *FucTA*, and *PGM1*.

### 3.8. RT-qPCR Validation of RNA-Seq Data

**Figure 8:**
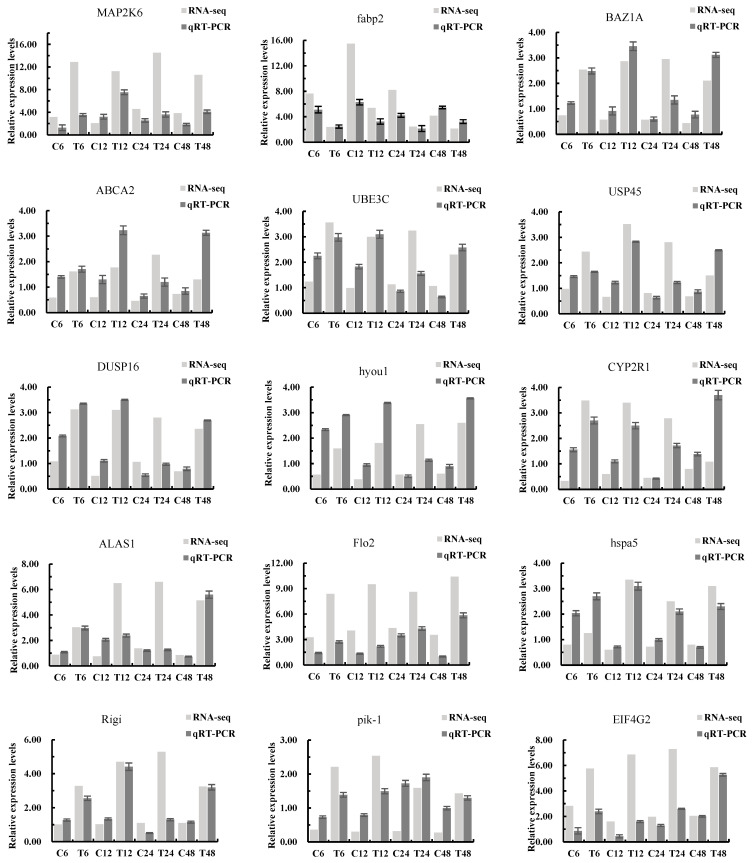
Real-time qPCR validation of RNA-seq profiles.

To validate RNA-Seq results, qRT-PCR was used to analyze the relative expression levels of 15 DEGs (Figure 8), including *MAP2K6*, *fabp2*, *BAZ1A*, *ABCA2*, *UBE3C*, *USP45*, *DUSP16*, *hyou1*, *CYP2R1*, *ALAS1*, *Flo2*, *hspa5*, *Rigi*, *pik-1*, and *EIF4G2*. The qRT-PCR results showed consistent expression trends with RNA-Seq data, confirming the reliability and accuracy of the transcriptomic analysis.

### 3.9. Data Quality Control and Screening of Metabolites

**Figure 9:**
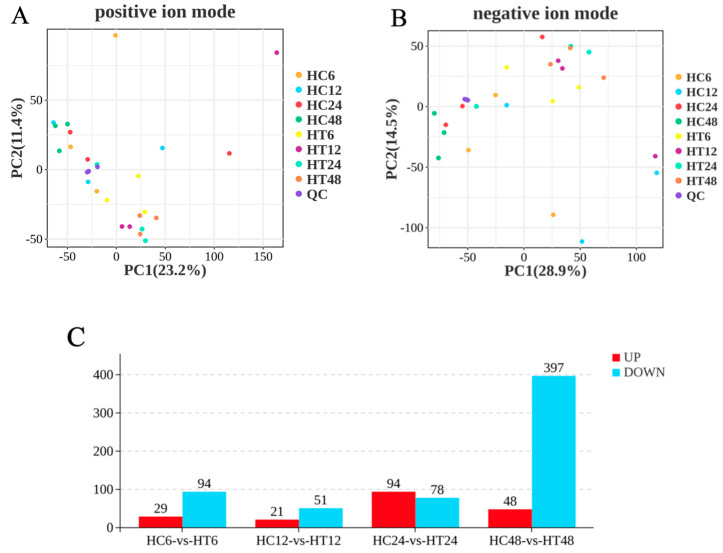
(**A**,**B**): Principal component analysis of QC samples in positive ion and negative ion modes. (**C**): Column chart of DEMs. Note: red and blue represent upregulated and downregulated DEMs.

**Figure 10:**
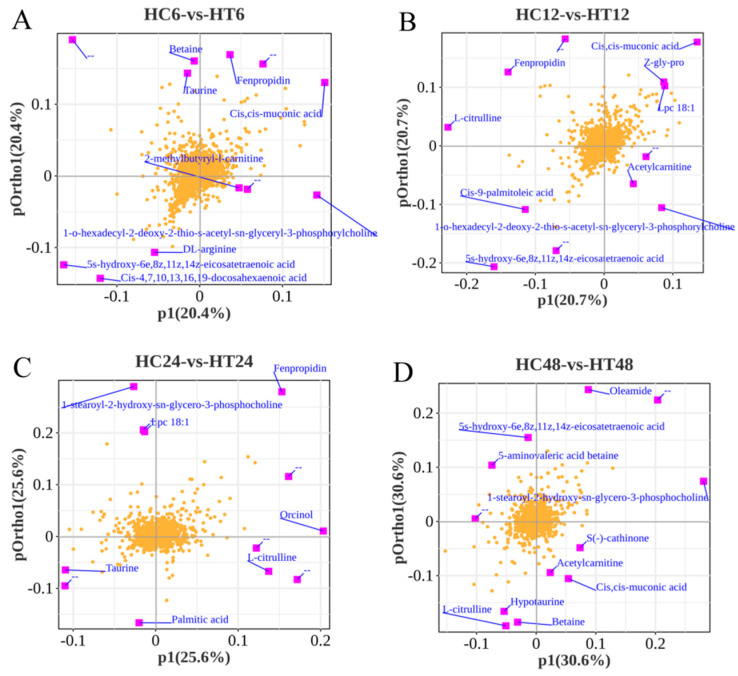
(**A**–**D**): Loadings of the orthogonal partial least squares discriminant analysis (OPLS-DA) for the four comparison groups.

Principal component analysis (PCA) of quality control (QC) samples showed tight clustering in both positive and negative ion modes, indicating high instrument stability, reliable data, and good biological reproducibility (Figure 9A,B). A total of 27,126 metabolites (POS+NEG) were identified. In the positive ion mode, 3706 known metabolites were annotated by matching secondary mass spectra (MS2), with 10,189 unannotated metabolites pending further analysis. In the negative ion mode, 2760 known metabolites were annotated, and 10,471 unannotated metabolites were retained (Table 4). DEMs were identified by combining variable importance in projection (VIP ≥ 1) from orthogonal partial least squares discriminant analysis (OPLS-DA) with a univariate *t*-test (*p* < 0.05). A total of 737 DEMs were obtained (Figure 9C), including 123 (29 upregulated, 94 downregulated) in HC6-vs-HT6, 72 (21 upregulated, 51 downregulated) in HC12-vs-HT12, 172 (94 upregulated, 78 downregulated) in HC24-vs-HT24, and 445 (48 upregulated, 397 downregulated) in HC48-vs-HT48. The higher number of downregulated vs. upregulated metabolites was consistent with transcriptomic results. Loading plots identified metabolites contributing most to metabolic pattern changes in HC6-vs-HT6, HC12-vs-HT12, HC24-vs-HT24, and HC48-vs-HT48 groups. Key contributors included cis-muconic acid, taurine, betaine, 2-Methylbutyryl-L-carnitine, and fenpropidin in HC6-vs-HT6 (Figure 10A); 5-HETE, LPC 18:1, Z-Gly-Pro, Cis-9-palmitoleic acid, and acetylcarnitine in HC12-vs-HT12 (Figure 10B); taurine, oleic acid, palmitic acid, and L-Citrulline in HC24-vs-HT24 (Figure 10C); and acetylcarnitine, Cis,cismuconic acid, betaine, hypotaurine, and 5-aminovaleric acid betaine in HC48 vs. HT48 (Figure 10D).

### 3.10. KEGG Enrichment Analysis of DEMs

**Figure 11:**
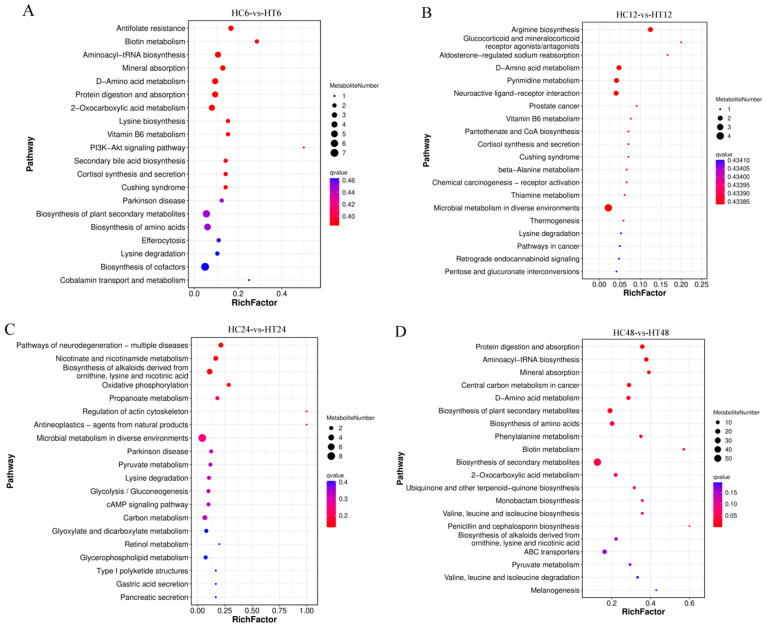
(**A**–**D**): KEGG enrichment analysis of the differential metabolites in the four comparison groups.

To characterize the biological processes involving DEMs across experimental groups, KEGG enrichment analysis was performed on DEMs from HC6-vs-HT6, HC12-vs-HT12, HC24-vs-HT24, and HC48-vs-HT48 comparisons, with the top 20 enriched pathways visualized in Figure 11. The 123 DEMs in HC6-vs-HT6 enriched 88 metabolism-related pathways, with significant enrichment in antifolate resistance, biotin metabolism, aminoacyl-tRNA biosynthesis, and D-amino acid metabolism (Figure 11A). The 72 DEMs in HC12-vs-HT12 enriched 47 pathways, including arginine biosynthesis, pyrimidine metabolism, biosynthesis of amino acids, and D-amino acid metabolism (Figure 11B). The 172 DEMs in HC24-vs-HT24 enriched 85 pathways, notably in sulfur metabolism, fatty acid biosynthesis, nucleotide metabolism, and ascorbate/aldarate metabolism (Figure 11C). The 445 DEMs in HC48-vs-HT48 enriched 78 pathways, predominantly in protein digestion/absorption, aminoacyl-tRNA biosynthesis, D-amino acid metabolism, mineral absorption, and phenylalanine metabolism (Figure 11D).

### 3.11. Integrated Transcriptomic and Metabolomic Analysis

**Figure 12:**
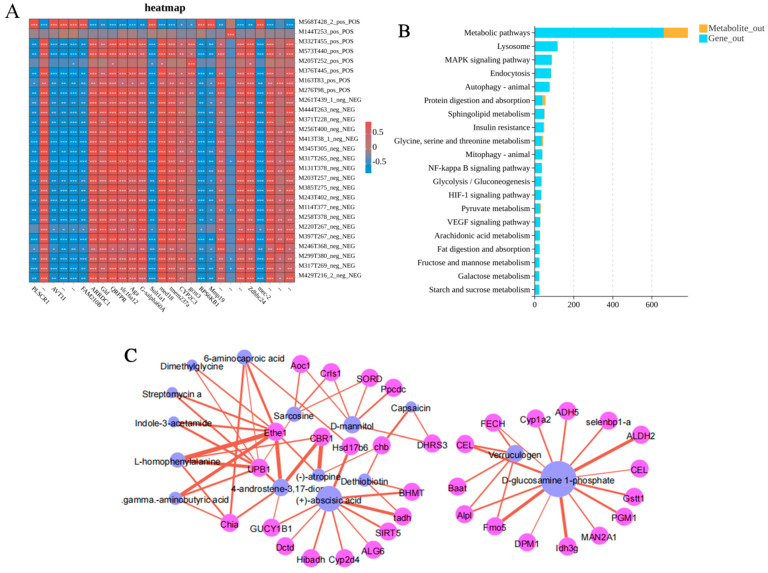
(**A**): Heatmap of correlation between DEGs and DEMs. Note: metabolites are displayed in columns, and genes are displayed in rows. Red indicates a positive correlation between DEGs and DEMs, while blue indicates a negative correlation. Asterisk (*, **, ***) denotes a statistically significant correlation between a DEG and DEM (*p* < 0.05, *p* < 0.01, *p* < 0.001). (**B**): Top 20 pathways co-enriched by DEGs and DEMs (*p* < 0.05). (**C**): Correlation network diagram of DEMs and DEGs in the top 20 enriched pathways.

To further elucidate the relationships between genes and metabolites in *C. antiquata* under ammonia nitrogen stress, integrated analysis was performed on 7823 DEGs and 737 DEMs. Figure 12A depicts a heat map, with gradient colors illustrating the correlation between DEGs and DEMs. Pearson correlation coefficient analysis revealed significant associations between expression levels of immune- and metabolism-related genes and metabolite concentrations. A total of 1341 DEGs and 132 DEMs were mapped to 197 shared KEGG pathways, with the top 20 pathways (*p* < 0.05) visualized in Figure 12B, including lysosome, MAPK signaling pathway, PI3K-Akt signaling pathway, Ras signaling pathway, Rap1 signaling pathway, and cAMP signaling pathway. These significantly enriched shared pathways were primarily associated with nutrient metabolism, detoxification, and oxidative stress, indicating their critical roles in *C. antiquata* responses to ammonia nitrogen stress.

To reveal the intrinsic associations between gene expression and metabolite changes in *C. antiquata* under ammonia nitrogen stress, we constructed a correlation network diagram using DEMs and DEGs from the top 20 enriched pathways (Figure 12C). This network contains 11 core metabolites (blue nodes) and 29 hub genes (pink nodes), forming interaction relationships through significant correlations, and overall presenting two functional subnetworks. Among them, (+)-abscisic acid is significantly positively correlated with *GUCY1B1* (cGMP signaling), *Hibadh* (energy metabolism), and *Cyp2d4* (xenobiotic metabolism), and GABA is strongly associated with the immune gene *Chia*, collectively participating in signal regulation and stress responses. D-glucosamine 1-phosphate is directly related to *MAN2A1* (glycosylation) and *ALDH2* (detoxification), while verruculogen analogs are significantly associated with selenbp1-a (antioxidation) and *ADH5* (detoxification), dominating metabolic remodeling and detoxification defense. The annotation information of the aforementioned genes is based on the KEGG database (https://www.genome.jp/kegg/, accessed on 1 January 2026), the Gene Ontology (GO) database (https://www.geneontology.org/, accessed on 1 January 2026), and the NCBI GenBank database.

## 4. Discussion

Ammonia nitrogen is a common water pollutant whose toxicity significantly affects the physiological metabolism and antioxidant system of aquatic organisms [49]. Ammonia nitrogen exerts toxic effects by interfering with ammonia metabolism, inducing oxidative stress, and disrupting the cell membrane structure [50,51]. In the toxicity test, the 48 h median lethal concentration (LC_50_-48 h) and safe concentration (SC) of ammonia nitrogen for *C. antiquata* were determined to be 99.06 mg/L and 9.91 mg/L, respectively. These values are relatively higher than those reported for other bivalve species, such as *Hyriopsis cumingii* (12.86 mg/L) [52], *Solenaia oleivora* (63.29 mg/L) [24], and *Spisula solidissima* (10.6 mg/L) [53], indicating that *C. antiquata* is less sensitive to ammonia nitrogen stress and exhibits greater tolerance. The safe concentration of ammonia nitrogen for *C. antiquata* (9.91 mg/L) is closely correlated with the actual ammonia nitrogen levels in agricultural aquaculture environments, which holds critical significance for guiding practical aquaculture management. Histopathological observations further revealed the target sites of ammonia nitrogen-induced damage: after 48 h of exposure to 8.915 mg/L unionized ammonia, extensive necrosis and detachment of hepatopancreatic epithelial cells occurred in *C. antiquata*, accompanied by hemocyte infiltration and vacuolation; gill tissues exhibited sparse arrangement of gill filaments, atrophy of connective tissues, and disintegration of columnar cell structures. These tissue-level damages not only directly confirm the toxic mechanism of ammonia nitrogen but also provide a physiological explanation for the abnormal behaviors and low tolerance of this species. The LC_50_-48 h and SC values established in this study provide critical benchmarks for ammonia nitrogen monitoring in aquaculture systems, enabling proactive management strategies to mitigate potential losses associated with ammonia toxicity in *C. antiquata* cultivation.

Environmental ammonia elevation triggers the generation of reactive oxygen species (ROS), including hydrogen peroxide (H_2_O_2_), superoxide anions (O^2−^), hydroxyl radicals (▪OH), and singlet oxygen (^1^O_2_), whose excessive accumulation disrupts metabolic homeostasis and threatens survival [54]. SOD and CAT act as critical antioxidant defenses against such oxidative damage, with their levels serving as key biomarkers of an organism’s anti-oxidative capacity [55,56,57]; prior studies have shown that ammonia can upregulate these enzymes [58]. The hepatopancreas, a key organ for metabolic detoxification in mollusks, is among the primary target organs for ammonia action [19,35]. In this study, SOD and CAT activities and MDA content in gill and hepatopancreas tissues of *C. antiquata* significantly increased at the initial stage (6 h) of ammonia stress, indicating rapid activation of antioxidant defense mechanisms against oxidative stress. This is consistent with Zhao’s findings on SOD/CAT activities in *Hyriopsis cumingii* hepatopancreas [52]. However, from 12 h to 48 h, these indices gradually decreased, even falling below control levels at 48 h. This may result from inhibited antioxidant enzyme synthesis or disrupted enzyme structure under prolonged stress, leading to reduced catalytic activity. Notably, the rapid increase in antioxidant enzyme activities is synchronously supported by transcriptomic data: at 6 h, the key encoding genes of SOD and CAT (e.g., *SOD1*, *SOD2*, *CAT*) were significantly upregulated in both tissues (log_2_ fold change > 1.5, *p* < 0.05). From 12 h to 48 h, these enzyme activities gradually decreased, even falling below control levels at 48 h; correspondingly, transcriptomic analysis revealed a continuous downregulation trend in the expression of SOD and CAT family genes during the same period. This correlation confirms that early activation of the antioxidant system is driven by the transcriptional induction of enzyme-encoding genes, which enhances the de novo synthesis of SOD and CAT to scavenge excessive reactive oxygen species (ROS) induced by ammonia stress. Ammonia nitrogen stress is highly toxic to aquatic organisms, and converting excess ammonia to Gln is a common detoxification mechanism. GLDH and GS are key enzymes in this process [59,60]. Both glutamate and Gln, being non-toxic and readily transported via the circulatory system, effectively detoxify endogenous ammonia from amino acid catabolism and exogenous ammonia [61,62]. In *Hyriopsis cumingii*, ammonia exposure initially stimulates GS activity in the hepatopancreas and gills, but subsequent tissue damage limits its detoxification capacity; meanwhile, GLDH levels in these tissues increase significantly over time [52]. Similarly, Wang et al. reported that ammonia stress elevated both GS activity and Gln content in *Apostichopus japonicus* [63]. In *C. antiquata*, ammonia exposure significantly increased GS activity and Gln content in gills and hepatopancreas, confirming Gln-mediated detoxification. GLDH activity surged early (6–24 h) to promote ammonia conversion, then declined at 48 h (but remained above baseline), potentially due to metabolic disorders [29,64,65]. Gill GS activity rose steadily, sustaining ammonia to Gln conversion to reduce free ammonia and maintain nitrogen balance [54], while hepatopancreatic Gln decreased at 48 h, likely from reduced GS activity and Gln utilization in stress-responsive pathways. Urea also plays a crucial role in ammonia detoxification, as evidenced by previous studies showing that aquatic animals with high ammonia tolerance efficiently convert ammonia into urea [66,67,68]. Overall, *C. antiquata* mitigates ammonia toxicity via a dual mechanism: converting ammonia to non-toxic Gln and urea.

RNA sequencing (RNA-seq), an effective tool for studying environmental adaptation, has been widely applied in stress response research of mollusks [24,35]. In this study, comparative transcriptomic analysis was performed on the hepatopancreas of *C. antiquata* in the control group (C) and ammonia nitrogen treatment group (T), yielding high-quality sequencing data: the Q20 and Q30 values of all samples were above 90%, indicating that the data reliability and integrity met the requirements for subsequent analyses. A total of 23,652 Unigenes were obtained after assembly, including 21,437 known genes (detection rate: 87.17%) and 2215 novel genes (accounting for 8.26% of the reference genome). These Unigenes were successfully annotated in multiple authoritative databases, such as NR, GO, KEGG, and eggNOG, providing abundant genetic resources for functional analysis. Differential expression analysis identified 7823 differentially expressed genes (DEGs), and cluster analysis showed that these DEGs could be clearly divided into two branches, corresponding to the control group and treatment group, indicating that ammonia nitrogen stress significantly reshaped the gene expression profile of the hepatopancreas in *C. antiquata*. In this study, DEGs in the hepatopancreas of *C. antiquata* under ammonia stress were significantly enriched in key signaling pathways. Activation of Toll-like receptor (TLR), NF-κB, and Toll-immune deficiency (Imd) signaling pathways indicated initiation of immune defense mechanisms. These pathways induce the expression of antimicrobial peptides and inflammation-related factors, protecting against pathogen invasion exacerbated by ammonia stress. Enrichment of NOD-like receptor (NLR) and tumor necrosis factor (TNF) signaling pathways further validated the importance of immune responses, synergistically regulating inflammation and cell survival with the aforementioned pathways. TLRs and NLRs, as pattern recognition receptors (PRRs), recognize pathogen-associated molecular patterns (PAMPs) or damage-associated molecular patterns (DAMPs) [69]. Upon environmental stress, these receptors sense endogenous danger signals released by cell damage, activating downstream NF-κB signaling [70,71]. Ammonia stress likely prompted similar danger signals in *C. antiquata* hepatopancreas, triggering NF-κB nuclear translocation to regulate proinflammatory cytokine genes, consistent with findings in *Corbicula fluminea* [35]. Activation of the TNF pathway may exacerbate the inflammatory cascade, disrupting cellular homeostasis via apoptosis or necrosis [72]. Co-activation of these pathways suggests overactivation of the immune system to counter environmental stress, though excessive inflammation may cause self-tissue damage. Changes in mitogen-activated protein kinase (MAPK) signaling and ubiquitin-mediated proteolysis reflect fine regulation of protein function and intracellular signaling under ammonia toxicity [73,74]. MAPK phosphorylates downstream transcription factors to modulate proliferation, differentiation, and stress responses, while ubiquitin-mediated proteolysis degrades misfolded proteins to maintain proteostasis and alleviate protein damage [75]. Additionally, enrichment of PPAR, insulin, and necroptosis pathways indicates that ammonia stress affects lipid/energy metabolism and programmed cell death. Abnormal regulation of these pathways may be the basis for functional disorders and cell death in the hepatopancreas under ammonia toxicity. Exploring the functional mechanisms of these pathways can further validate them as a universal biomarker combination for health monitoring in multi-species aquaculture. This approach also reduces reliance on time-consuming traditional physiological tests (such as enzyme activity assays), thereby enhancing the efficiency of aquaculture management. Additionally, investigating the genetic basis of ammonia tolerance will facilitate the development of stress-resistant strains.

Metabolomic analysis visually revealed physiological state changes in *C. antiquata* under ammonia nitrogen stress, with key contributing metabolites and enriched pathways in different treatment groups providing critical clues to toxic mechanisms. Among antioxidant and detoxification-related metabolites, taurine was a major contributor in multiple groups (HC6-vs-HT6, HC24-vs-HT24). Taurine exerts functions in antioxidant activity, osmoregulation, and free radical scavenging [76]. Its significant elevation indicated that *C. antiquata* attempted to mitigate oxidative stress damage and maintain intracellular homeostasis by increasing taurine levels under ammonia stress. The presence of cis-muconic acid, possibly associated with phenylalanine metabolism, suggested activation of this pathway as an intermediate product, implying metabolic conversion for detoxification [20]. Changes in betaine levels in HC6-vs-HT6 and HC48-vs-HT48 reflected its roles in osmotic regulation and methyl group metabolism, supplying methyl groups for biosynthesis [77]. The significant variation of L-citrulline in HC24-vs-HT24 corresponded to enrichment of the arginine biosynthesis pathway. As a precursor of arginine, L-citrulline accumulation suggested that *C. antiquata* aimed to convert excess ammonia into urea or other nitrogenous compounds via arginine biosynthesis for excretion, thereby reducing ammonia toxicity [78]. Transcriptomic data revealed that genes encoding metabolites such as arginine (e.g., *ass1*, *ARG2*) were upregulated synchronously during ammonia stress, indicating that the synthesis pathway of arginine and other metabolites was activated at the transcriptional level under ammonia stress, thereby promoting ammonia transformation.

Integrated analysis of transcriptomic and metabolomic data provides a comprehensive view of molecular response mechanisms in *C. antiquata* under ammonia nitrogen stress. In the context of crosstalk between signaling and metabolic pathways, activation of NF-κB and MAPK signaling pathways likely regulates the expression of genes involved in amino acid and fatty acid metabolism. NF-κB activation suppresses fatty acid synthesis-related genes [79], consistent with fatty acid content changes observed in metabolomics. The MAPK pathway modulates glycolysis and fatty acid β-oxidation enzymes via phosphorylation, influencing levels of energy metabolites like acetylcarnitine [80,81]. Metabolite-signaling pathway associations reveal tight links between taurine/betaine variations and enrichment of sulfur/glycine metabolism pathways in transcriptomics [76]. As a sulfur metabolism product, taurine not only exhibits antioxidant activity but may also feedback-regulate MAPK phosphorylation to alleviate oxidative stress damage [73,75]. Betaine accumulation, linked to glycine metabolism activation, collaborates in osmotic regulation [77]. In the association analysis of transcriptome and metabolome, inconsistency between transcription and metabolic trends was found, which was due to the dynamic regulation of the ammonia detoxification process through hierarchical regulation (transcription→enzyme activity→metabolic flow) and pathway crosstalk, rather than simple one-to-one correspondence between genes and metabolites. In summary, integrated omics analysis demonstrates that *C. antiquata* counters ammonia stress through immune defense activation, signal transduction regulation, and metabolic network remodeling. This study focuses on the hepatopancreas to elucidate molecular response mechanisms under ammonia nitrogen stress, as the hepatopancreas is the core organ responsible for ammonia nitrogen detoxification and metabolism in *C. antiquata*; phenotypic and physiological responses of the gill tissue have been clarified in previous experiments. Future research can further conduct omics analysis of gill tissue to improve comprehensive mechanism analysis of “ammonia exchange (gill)—internal detoxification (hepatopancreas)”, and more comprehensively reveal the adaptive strategies of *C. antiquata* to ammonia nitrogen stress.

## 5. Conclusions

In summary, this study systematically investigated the toxic effects and multifaceted response mechanisms of *C. antiquata* under ammonia nitrogen stress. In coping with ammonia nitrogen stress, *C. antiquata* resists oxidative injury through the rapid activation of antioxidant enzymes (SOD, CAT) in the gills and hepatopancreas, and concomitantly accomplishes ammonia detoxification through a dual mechanism involving Gln and urea synthesis. Transcriptomic and metabolomic analyses of hepatopancreas identified 7823 DEGs and 737 DEMs, both associated with immune and metabolic functions. DEGs enriched pathways mainly included metabolic pathways, NF-kappa B signaling pathway, RIG-I-like receptor signaling pathway, NOD-like receptor signaling pathway, Toll and Imd signaling pathway, and so on. DEMs are mainly involved in protein digestion/absorption, aminoacyl-tRNA biosynthesis, D-amino acid metabolism, mineral absorption, phenylalanine metabolism, and so on. The “gene-metabolite” network constructed using Pearson correlation analysis revealed that core metabolites, including γ-aminobutyric acid (GABA) and D-glucosamine 1-phosphate, had strong associations with the immune gene *Chia*, glucose metabolism gene *MAN2A1*, and detoxification gene *ALDH2*, respectively. Additionally, NF-κB signaling genes (e.g., *Tbk1*) were co-expressed with steroid and amino acid metabolites, which collectively mediated the antioxidant response, nitrogen metabolism regulation, and immune defense response of *C. antiquata* under ammonia nitrogen stress, providing multi-omics association evidence for elucidating the molecular mechanism of its ammonia nitrogen tolerance. This study represents the first multi-omics analysis of *C. antiquata’s* response to ammonia stress, providing a new theoretical foundation for research on aquatic organisms’ environmental stress responses, offering scientific support for formulating ammonia pollution control strategies in bivalve aquaculture, and delivering tangible practical value for the sustainable development of aquaculture. Future research will focus on validating these biomarkers in field settings and exploring the genetic basis of ammonia tolerance to facilitate the cultivation of stress-resistant strains.

## Figures and Tables

**Table 1:** Primer sequences used for RT-qPCR verification.

Gene Name	Gene Id	Primer Sequence (5′-3′)	Tm (°C)
MAP2K6	ACF0H5_001111	F: TGCTGCCTCTCCTACTCCAT	60
		R: CTTGCTCTTTCTGCCACCGA	60
fabp2	ACF0H5_001391	F: ACGAGTGTGTCCCCAAGCAT	61
		R: CCCATTTGTTTCCTTCAAGGGTAGC	62
BAZ1A	ACF0H5_002977	F: GTTGTGAGAATGAAGATGGAGCA	59
		R: GGTGGGGGACCAGCAAAAAT	60
ABCA2	ACF0H5_004216	F: AGCATTGATTGGTCATCCACCT	60
		R: AACCGAGCGTCCATCTTTGA	59
UBE3C	ACF0H5_005470	F: CAGGTTTAGATGAGGCTGGC	58
		R: TCTGACTGAAGAATCCACGGTT	59
USP45	ACF0H5_007893	F: TGGAGAATGTCAAAACTGTGCT	60
		R: CCCCACTTGTAGCCCCTTC	59
DUSP16	ACF0H5_008455	F: ACCCCTGACACACAAAGTGC	60
		R: AGGTGCTATCGGAGACAACG	60
hyou1	ACF0H5_012287	F: CAGGAAGATGACACGCCCAA	59
		R: AGTGCTGCTAATGCCTCTGG	60
CYP2R1	ACF0H5_012607	F: GGAATGGCAGAAAGCGTATGG	59
		R: TGCTCACAGGTCACAAACATCT	60
ALAS1	ACF0H5_014142	F: CGTGATGGTTGTATGGAGAAAATGG	60
		R: TGTTGTAGGTGGTAATGATGTAGTG	58
Flo2	ACF0H5_017347	F: TGCTCAGTTGGTTAGGGAAGT	58
		R: ACATTGGCAGTTTGGGCTCT	60
hspa5	ACF0H5_021214	F: ATGGAAAGGAGCCAAGTCGT	59
		R: TCACATCTTCAACCCGTCCC	59
Rigi	ACF0H5_008989	F: CTGCTATGGAGGGTGGTTCC	59
		R: GGCTACCGATGTGGCAATGA	60
pik-1	ACF0H5_013956	F: GACCATCAAGGGGCTTCCTC	60
		R: CGGCTCCATACTCTTCAACCA	59
EIF4G2	ACF0H5_015485	F: GTCCACGACCTTCAATGGCT	60
		R: AAGTCACCAAACGCACCAGT	60
β-actin		F:CTGTGCTACGTTGCCCTGGACTT	61
		R:TGGGCACCTGAATCGCTCGTT	62

**Table 2:** Summary table of sample sequencing data.

Sample	Raw Reads	HQ Reads (%)	Valid Reads (%)	Raw Data (bp)	Clean Data (bp)	Q20 (%)	Q30 (%)	GC (%)
C6-1	42,077,668	42,046,102 (99.92%)	41,051,244 (97.63%)	6,311,650,200	6,217,794,976	98.97	97.27	39.59
C6-2	45,203,040	45,164,432 (99.91%)	44,080,028 (97.60%)	6,780,456,000	6,654,763,463	98.95	97.23	39.13
C6-3	37,744,484	37,714,852 (99.92%)	36,502,468 (96.79%)	5,661,672,600	5,570,137,076	98.73	96.74	38.88
C12-1	38,859,422	38,817,366 (99.89%)	37,937,542 (97.73%)	5,828,913,300	5,730,504,850	98.49	96.01	39.23
C12-2	36,006,152	35,958,196 (99.87%)	34,973,948 (97.26%)	5,400,922,800	5,304,999,593	98.61	96.39	39.99
C12-3	41,795,880	41,735,142 (99.85%)	40,842,384 (97.86%)	6,269,382,000	6,178,840,447	98.67	96.60	39.34
C24-1	41,534,818	41,475,742 (99.86%)	40,599,142 (97.89%)	6,230,222,700	6,132,197,327	98.62	96.52	39.53
C24-2	41,788,190	41,755,176 (99.92%)	40,591,040 (97.21%)	6,268,228,500	6,147,945,589	98.58	96.41	39.41
C24-3	39,415,646	39,353,426 (99.84%)	38,446,718 (97.70%)	5,912,346,900	5,827,220,696	98.61	96.45	39.40
C48-1	45,990,294	45,944,402 (99.90%)	44,764,590 (97.43%)	6,898,544,100	6,756,482,348	98.58	96.43	39.50
C48-2	39,879,816	39,822,530 (99.86%)	38,591,816 (96.91%)	5,981,972,400	5,875,074,053	98.63	96.50	39.52
C48-3	42,359,264	42,295,048 (99.85%)	40,906,448 (96.72%)	6,353,889,600	6,240,454,496	98.72	96.70	39.05
T6-1	43,122,852	43,087,622 (99.92%)	41,901,810 (97.25%)	6,468,427,800	6,383,421,902	98.70	96.68	38.54
T6-2	40,655,734	40,615,242 (99.90%)	39,528,416 (97.32%)	6,098,360,100	5,962,594,745	98.75	96.78	39.29
T6-3	39,171,450	39,122,930 (99.88%)	38,001,978 (97.13%)	5,875,717,500	5,746,211,960	98.75	96.78	39.07
T12-1	38,606,722	38,549,740 (99.85%)	37,838,738 (98.16%)	5,791,008,300	5,668,330,228	98.50	96.14	39.18
T12-2	44,569,348	44,512,896 (99.87%)	43,716,368 (98.21%)	6,685,402,200	6,539,309,098	98.45	96.05	38.82
T12-3	44,270,568	44,207,514 (99.86%)	43,338,138 (98.03%)	6,640,585,200	6,500,723,105	98.67	96.56	38.68
T24-1	37,084,030	37,034,852 (99.87%)	36,229,286 (97.82%)	5,562,604,500	5,416,045,636	98.04	95.18	39.25
T24-2	36,707,064	36,647,148 (99.84%)	35,818,740 (97.74%)	5,506,059,600	5,425,354,659	98.65	96.59	39.08
T24-3	43,085,074	43,026,490 (99.86%)	41,963,348 (97.53%)	6,462,761,100	6,341,920,408	98.51	96.13	38.38
T48-1	44,096,458	44,010,468 (99.80%)	42,796,362 (97.24%)	6,614,468,700	6,451,747,959	98.80	96.93	39.28
T48-2	39,263,894	39,195,218 (99.83%)	38,058,770 (97.10%)	5,889,584,100	5,763,911,662	98.67	96.62	39.42
T48-3	39,418,848	39,353,196 (99.83%)	38,449,118 (97.70%)	5,912,827,200	5,778,913,401	98.52	96.31	39.25
Total	982,706,716	981,445,730 (99.87%)	956,928,440	147,406,007,400	144,614,899,677			

Note: raw data—number of sequencing raw reads. HQ reads—number, and percentage of high-quality reads (based on raw reads). Valid reads—the number and percentage of reads (based on HQ reads) that do not match the ribosomal RNA. Raw data (bp)—total number of bases of sequencing data. Clean data (bp)—total number of bases in filtered high-quality data. Q20 (%)—number of bases with sequencing base quality values of Q20 or higher and percentage of clean data. Q30 (%)—number of bases with sequenced base quality values at Q30 or higher level and percentage of clean data. GC (%)—proportion of filtered sequence base GCs.

**Table 3:** Comparison reference genome statistics.

Sample	Valid Reads	Unmapped (%)	Unique_Mapped (%)	Multiple_Mapped (%)	Total_Mapped (%)
C6-1	41,051,244	8,497,520 (20.70%)	31,107,722 (75.78%)	1,446,002 (3.52%)	32,553,724 (79.30%)
C6-2	44,080,028	8,528,970 (19.35%)	34,018,537 (77.17%)	1,532,521 (3.48%)	35,551,058 (80.65%)
C6-3	36,502,468	8,653,876 (23.71%)	26,761,276 (73.31%)	1,087,316 (2.98%)	27,848,592 (76.29%)
C12-1	37,937,542	6,710,958 (17.69%)	29,805,999 (78.57%)	1,420,585 (3.74%)	31,226,584 (82.31%)
C12-2	34,973,948	5,196,752 (14.86%)	28,161,821 (80.52%)	1,615,375 (4.62%)	29,777,196 (85.14%)
C12-3	40,842,384	8,120,092 (19.88%)	31,132,106 (76.22%)	1,590,186 (3.89%)	32,722,292 (80.12%)
C24-1	40,599,142	8,205,724 (20.21%)	30,852,309 (75.99%)	1,541,109 (3.80%)	32,393,418 (79.79%)
C24-2	40,591,040	7,525,598 (18.54%)	31,557,864 (77.75%)	1,507,578 (3.71%)	33,065,442 (81.46%)
C24-3	38,446,718	8,160,668 (21.23%)	28,833,744 (75.00%)	1,452,306 (3.78%)	30,286,050 (78.77%)
C48-1	44,764,590	8,700,077 (19.44%)	34,365,333 (76.77%)	1,699,180 (3.80%)	36,064,513 (80.56%)
C48-2	38,591,816	6,348,215 (16.45%)	30,730,234 (79.63%)	1,513,367 (3.92%)	32,243,601 (83.55%)
C48-3	40,906,448	7,818,194 (19.11%)	31,749,379 (77.61%)	1,338,875 (3.27%)	33,088,254 (80.89%)
T6-1	41,901,810	9,022,091 (21.53%)	31,616,580 (75.45%)	1,263,139 (3.01%)	32,879,719 (78.47%)
T6-2	39,528,416	6,829,720 (17.28%)	31,316,835 (79.23%)	1,381,861 (3.50%)	32,698,696 (82.72%)
T6-3	38,001,978	8,089,150 (21.29%)	28,573,197 (75.19%)	1,339,631 (3.53%)	29,912,828 (78.71%)
T12-1	37,838,738	7,158,325 (18.92%)	29,371,978 (77.62%)	1,308,435 (3.46%)	30,680,413 (81.08%)
T12-2	43,716,368	8,908,013 (20.38%)	33,488,026 (76.60%)	1,320,329 (3.02%)	34,808,355 (79.62%)
T12-3	43,338,138	8,849,992 (20.42%)	33,107,733 (76.39%)	1,380,413 (3.19%)	34,488,146 (79.58%)
T24-1	36,229,286	6,772,182 (18.69%)	28,208,223 (77.86%)	1,248,881 (3.45%)	29,457,104 (81.31%)
T24-2	35,818,740	6,594,220 (18.41%)	28,063,458 (78.35%)	1,161,062 (3.24%)	29,224,520 (81.59%)
T24-3	41,963,348	8,722,939 (20.79%)	31,927,271 (76.08%)	1,313,138 (3.13%)	33,240,409 (79.21%)
T48-1	42,796,362	6,690,819 (15.63%)	34,602,886 (80.85%)	1,502,657 (3.51%)	36,105,543 (84.37%)
T48-2	38,058,770	6,363,583 (16.72%)	30,248,454 (79.48%)	1,446,733 (3.80%)	31,695,187 (83.28%)
T48-3	38,449,118	6,987,707 (18.17%)	29,980,548 (77.97%)	1,480,863 (3.85%)	31,461,411 (81.83%)

Note: unmapped (%), number of reads, and percentage of valid reads that were not matched to the reference genome. Unique_Mapped (%), number of reads, and percentage of valid reads for the reference genome with the unique match. Multiple_Mapped (%), the number of reads on the reference genome and the percentage of valid reads. Total_Mapped (%)—the number of reads that can be localized to the genome and the percentage of valid reads.

**Table 4:** Identification of metabolites in positive ion and negative ion modes.

Type	All	Known	Unknown
POS	13,895	3706	10,189
NEG	13,231	2760	10,471

## Data Availability

Transcriptome data were uploaded to the NCBI SRA database (PRJNA1303244).

## Supplementary Materials

The following supporting information can be downloaded at: https://www.mdpi.com/article/10.3390/ani16020192/s1, Table S1: 66 differential expression DEGs across all time points.
